# Survival of infants born at periviable gestation: The US national database

**DOI:** 10.1016/j.lana.2022.100330

**Published:** 2022-07-25

**Authors:** Ibrahim Qattea, Mohsen A.A. Farghaly, Mohammad O. Kattea, Nibras Abdula, Mohamed A. Mohamed, Hany Aly

**Affiliations:** a Cleveland Clinic Children's, Cleveland, OH, USA; b Nassau University Medical Center, New York, USA; c Aswan Faculty of Medicine, Egypt; d University of Edinburgh, UK; e Al-Baath University, Syria

**Keywords:** aOR, adjusted odds ratio, CI, confidence interval, HCUP, Healthcare Cost and Utilization Project, ICD, International Classification of Diseases, LOS, length of stay, NIS, National Inpatient Sample

## Abstract

**Background:**

Substantial differences exist in the approach to resuscitating infants born at periviable gestation. Evaluation of current survival may help guide prenatal counselling and provide accurate expectations of clinical outcomes. We aimed to assess the US national survival trends in periviable infants born at gestational age (GA) ≤24 weeks.

**Methods:**

We used de-identified patient data obtained from the US Healthcare Cost and Utilization Project (HCUP) from 2007 to 2018. All infants with documented GA ≤24 weeks were included. The Cochran-Armitage test was used for trend analyses. Regression analyses were conducted for variables associated with survival.

**Findings:**

A total of 44,628,827 infant records were identified with 124,345 (0.28%) infants born ≤24 weeks; of those, 77,050 infants <24 weeks and 47,295 infants had completed 24 weeks. Survival rates for infants <24 weeks and with completed 24 weeks were 15.4% and 71.6%, respectively, with higher survival over the years (*Z* = 9.438, *P*<0.001 & *Z* = 3.30, *P*<0.001, respectively). Survival was lower in males compared to females (aOR = 0.96, CI: 0.93–0.99 & aOR = 0.94, CI: 0.92–0.96, respectively) and with private insurance compared to public insurance (aOR = 0.74, CI: 0.71–0.77 & aOR = 0.67, CI: 0.65–0.69, respectively). Survival was higher when birth weight was >500 g compared to ≤500 g (aOR = 4.62, CI:3.23–5.02 & aOR = 5.44, CI: 4.59–5.84, respectively). Black (aOR = 1.33, CI: 1.31–1.36 & aOR = 1.24, CI: 1.20–1.32, respectively) and Hispanic (aOR = 1.29, CI: 1.27–1.32 & aOR = 1.27, CI: 1.22–1.30, respectively) had higher survival than White.

**Interpretation:**

There is a national increase in survival over the years in infants born at periviable GA. BW >500 is associated with >4 folds higher survival compared to ≤500 g. The results of this study should be cautiously interpreted as long-term outcomes are unknown

**Funding:**

This research did not receive any specific grant from funding agencies in the public, commercial, or not-for-profit sectors.

**Research in context**

**Evidence before this study**

Premature birth continues to be a leading cause of infant mortality in the US. Premature infants are considered “periviable” when born at a gestational age (GA) between 20 0/7 and 25 6/7 weeks. There is a wide variation in the reported survival of periviable infants born at 22 weeks (0–37%), 23 weeks (1–64%), and 24 weeks (31–78%).

**Added value of this study**

During the study years 2007–2018, survival of infants born at GA <24 weeks increased from 18.4% to 31.9% and for infants born with completed 24 weeks, increased from 68.4% to 73.3%. When birth weight (BW) is greater than 500 g, the odds of survival in these infants increases by four to five folds. Female sex, ethnic minority and public insurance are all associated with increased survival.

**Implications of all the available evidence**

In the US, there is a national increase in survival of infants born at GA ≤24 weeks. In addition to BW >500 g, several factors are associated with improved survival of periviable newborns.

Alt-text: Unlabelled box

## Introduction

According to the National Institute of Child Health and Human Development (NICHD), the Section on Perinatal Pediatrics of the American Academy of Pediatrics, and the American College of Obstetricians and Gynecologists, a periviable birth is defined when delivery occurs from 20 0/7 weeks to 25 6/7 weeks of gestation.^1^ Care of periviable infants remains an ongoing challenge in the field of neonatal and perinatal medicine. These infants contribute significantly to the burden of neonatal morbidity and mortality and long-term neurodevelopmental disability.^2^ Substantial differences in approach to resuscitation and management of these infants are observed across centres.^3^ The NICHD network reported an improved survival over the last few years for infants born at 22 to 24 weeks of gestation from 30% to 36%.^3^

Providing national benchmarks for survival is critical for managing caregivers and health legislators. A few reports showed a wide variation in survival rates at periviable gestational ages, ranging from 0% to 37% at 22 weeks, 1–64% at 23 weeks, 31–78% at 24 weeks, and 59–86% at 25 weeks of gestation,^4^ The decision of families and health care teams to resuscitate infants born at the limit periviability is not an easy task. Several ethical and clinical factors are considered when making such decisions. The availability of established benchmarks for the survival of these infants is critical to ensure providing accurate estimates to the decision-makers. The reports mentioned above are, to some extent, helpful in providing outcome data to caregivers and families, although they do not necessarily reflect the national benchmark values as they typically originate from large academic urban facilities. Therefore, there is an unmet need to establish a national benchmark for survival rates for infants born at the limit of viability. It is also essential to find out whether the higher survival reported by NICHD is restricted to large academic centres or is nationally generalizable. This study aimed to assess the national survival rate for infants born at ≤ 24 weeks of gestation and the survival trends over time during the last few years. We hypothesized that there is a higher trend for survival of infants born ≤ 24 weeks of gestation during the study period. The study used the Healthcare Cost and Utilization Project (HCUP) from January 1^st^ 2007 through December 31^st^ 2018. This dataset reflects all inpatients in the US.

## Methods

### Data sources and management

The study used de-identified patient data produced by the Healthcare Cost and Utilization Project (HCUP) from the Agency for Healthcare Research and Quality (AHRQ).^5^ HCUP contains the biggest collection of hospital discharge data in the United States (US). HCUP produces the National Inpatient Sample (NIS) dataset annually, including 20% of the HCUP samples and weighted to reflect the entire national data. Each year more than seven million cases are drawn from thousands of hospitals across the US with various care levels (primary-tertiary), types of insurance (public or private), size of the hospital (small, medium, or large), and many other demographic and clinical characteristics. HCUP used (ICD-9-CM) diagnosis and procedure codes until October 2015 and (ICD-10-CM) codes from the last three months of 2015 through 2018. The NIS is designed as a random sample of all US community hospitals from states that contribute their State Inpatient Databases to the HCUP. Data elements in the NIS are constructed in a uniform format with quality checks in place. NIS data are available annually, thereby allowing analysis of trends over time. The unweighted data contains more than 7 million hospital stays each year, whereas weighted data estimates more than 35 million hospitalizations nationally. The current study utilized the weighted data that exhaustively represented all 35 million patients admitted during the study period.

### Study design and population

The study included all newborn inpatients with postnatal age ≤28 days from January 1^st^ 2007 through December 31^st^ 2018. Infants diagnosed with gestational age (GA) less than 24 weeks (23 6/7 weeks and less) and completed 24 weeks (24 0/7 through 24 6/7 weeks) were identified. Infants transferred to another healthcare facility would have two records; one record at the birthing hospital and another record at the referral hospital. To avoid duplication of records, we excluded all transported out records/infants and included all transported in records/infants. Of note, any infant born with heartbeats is considered an admission and consequently is expected to be included in the dataset regardless of resuscitation efforts or disposition to the neonatal ICU. However, stillbirths are not included in the dataset. The study identified essential infant demographic factors: birth weight (BW), gender, and race, as well as major system-based social exposures including insurance type, and median household income. *Supplementary Table* S1 shows ICD-9 and ICD-10 codes used for the study.

The primary outcome of this study was survival of infants defined in the HCUP dataset as survival until hospital discharge or transfer to another facility. Hospital survivals (%) were stratified by gender, race/ethnicity, BW category (≤500 g and >500 g), and type of insurance. Multivariate logistic regression analysis was conducted for factors associated with survival. Two separate regression models were constructed; one for infants with GA <24 weeks and another for infants with completed 24 weeks. Variables included in the regression analyses were essential infant demographics (sex, ethnicity/ race, and BW category) and two system-based social exposures (type of insurance and median household income). We identified two major comorbidities known to hinder the long-term outlook of periviable infants; severe intraventricular haemorrhage (IVH) and necrotizing enterocolitis (NEC). Severe IVH is diagnosed when bleeding in the brain occupies >50% of the lateral ventricles with associated ventricular dilatation (grade 3) or if the blood extravasate into brain tissue (grade 4).^6^ NEC is defined when an infant has feeding intolerance associated with abdominal distension, systemic signs and radiographic signs.^7^

Trend analyses were conducted for the annual prevalence of survival and morbidities during the years 2007–2018 stratified by GA, BW category, and sex using the Cochran-Armitage (CA) test for ordered alternatives. *P*-value was calculated to decide whether to accept or reject the null hypothesis for each trend. All analyses were performed using SPSS 25 statistical software (SPSS Inc., Chicago, IL). This study used weighted data for all analyses.

## Results

During the study period (January 1, 2007 to December 31, 2018), 44,628,827 newborns were identified; 641,601 of them were excluded due to major congenital/chromosomal anomalies and being transferred to other health facilities. A total of 124,345 (0.28%) infants were born at GA ≤ 24 weeks, of those, 77,050 infants were born <24 weeks, and 47,295 were born at 24 weeks of gestation. There was a disproportionately high representation of Black infants born at periviable age. The ethnic distribution of the study population was 38,201 White, 34,158 Black, 18,939 Hispanic, and 33,047 others. Mortality among all included newborns (*n* = 43,987,226) was 182,978 (0.41%) and mortality among all periviable infants was 71,940 (57.86%).

Table 1 describes the demographic and clinical characteristics of the study population. In the group of infants with GA <24 weeks (*N* = 77,050), only 18,533 (24.1%) survived and in infants born with completed 24 weeks of gestation (*n* = 47,295), a total of 33,872 (71.6%) survived. Survival among males was lower than females in infants <24 weeks (*p* < 0.001) and completed 24 weeks (*p* < 0.001) Survival differed significantly according to race in <24 weeks (*p* < 0.001) and completed 24 weeks (*p* < 0.001) populations. Infants with private insurance had significantly lower survival than public insurance in <24 weeks (*p* < 0.001) and completed 24 weeks (*p* < 0.001) groups.

**Table 1:** Factors influencing survivability in infants born ≤ 24 weeks of gestation.

		Preterm infants < 24 weeks, *N* = 77,050			Preterm infants 24 weeks, *N* = 47,295		
		Total	Alive	*P*-value	Total	Alive	*P*-value
Gender	Female	34,939	9124 (26.1)	*P*<0.001	25,480	17,635 (69.2)	*P*<0.001
	Male	41,768	9409 (22.5)		21,766	16,213 (74.5)	
Race/Ethnicity	White	22,834	5129 (22.5)	*P*<0.001	15,367	10,868 (70.7)	*P*<0.001
	Black	21,632	5809 (26.9)		12,526	9362 (74.7)	
	Hispanic	11,801	3173 (26.9)		7138	5340 (74.8)	
	Others	20,783	4424 (21.3)		12,264	8301 (67.7)	
Primary expected payer	Public insurance	37,434	10,607 (28.3)	*P*<0.001	26,673	19,602 (73.5)	*P*<0.001
	Private insurance	28,728	6326 (22)		16,612	11,700 (70.4)	
	Others	10,889	1600 (14.7)		4011	2570 (64.1)	
Median household income national quartile for patient's zip code ($)	0–25%	27,011	6706 (24.8)	*P*<0.001	16,462	11,959 (72.6)	*P*<0.001
	26–50%	18,603	4577 (24.6)		11,963	8543 (71.4)	
	51–75%	17,113	4017 (23.5)		10,466	7392 (70.6)	
	76–100%	13,103	2902 (22.2)		7536	5419 (71.9)	
Birth weight (g)	≤500	36,392	2698 (7.4)	*P*<0.001	43,772	32,283 (45.1)	*P*<0.001
	> 500	40,659	15,836 (38.9)		3523	1590 (73.8)	

Data are expressed in numbers (%). All numbers represent weighted data. Chi-square test was used to compare categories.

Survival significantly differed according to the infant's BW category. When combining the two GA variables (<24 weeks and completed 24 weeks) and the two BW variables (≤500 g and >500 g), four categories of infants were recognized: infants (< 24 weeks and ≤500 g), infants (<24 weeks and >500 g), infants (completed 24 weeks and ≤500 g), and infants (completed 24 weeks and >500 g). The per cent survival for these four groups were 7.4%, 38.9%, 45.1%, and 73.8%, respectively.

Logistic regression analyses for factors associated with survival in infants born with GA < 24 weeks were as follows. Male sex was associated with lower odds for survival (0.96, 95% CI 0.93–0.99, *p*< 0.001). Infants of a minority race (Black and Hispanic) had higher odds of survival. Compared to White, aOR for survival for Blacks was 1.33 (1.31–1.36), *p* < 0.001 and for Hispanics was 1.29 (1.27–1.32), *p*< 0.001. Survival was lower when infants were insured with private payer compared to public insurance, aOR = 0.74, 95% CI 0.71–0.77, *p*< 0.001. Survival differed significantly according to median household income national quartile for patient's zip code. Compared to the group of infants in the lowest quartile of income ≤25%, infants with a household income 26–50% had survival aOR = 1.28, 95% CI 1.15–1.31, *p*< 0.001, infants with a household income 51–75% had survival aOR = 0.87, 95% CI 0.84–0.91, *p*< 0.001, and infants with a household income >75% had survival aOR = 0.94, 95% CI 0.90–0.98, *p*< 0.001. Survival was higher in infants with BW >500 g compared to infants with BW≤500, aOR = 4.62, 95%CI: 3.23–5.02, *p* < 0.001.

Logistic regression analyses for factors associated with survival in infants born with GA of completed 24 weeks were as follows. Male sex was associated with lower odds for survival, aOR = 0.94, CI 0.92–0.96, *p* <0.001. Infants of a minority race (Blacks and Hispanics) had higher odds of survival. Compared to White, aOR for survival in Blacks was 1.24, 95%CI 1.20–1.32, *p* <0.001 and in Hispanics was 1.27, 95%CI 1.22–1.30, *p* <0.001. Survival was lower when infants were insured with private payer compared to public insurance, aOR = 0.67, 95%CI 0.65–0.69, *p* <0.001. Survival differed significantly according to median household income national quartile for patient's zip code. Compared to the group of infants in the lowest quartile of income ≤25%, infants with household income 26–50% had survival aOR = 0.98, 95%CI 0.96–0.99, *p* <0.001, infants with household income 51–75% had survival aOR = 0.95, 95%CI 0.92–0.98, *p* <0.001, and infants with household income >75% had survival aOR = 0.86, 95%CI 0.83–0.89, *p* <0.001. Survival was higher in infants with BW >500 g compared to infants with BW≤500, aOR = 5.44, 95%CI 4.59–5.84, *p* <0.001.

**Figure 1:**
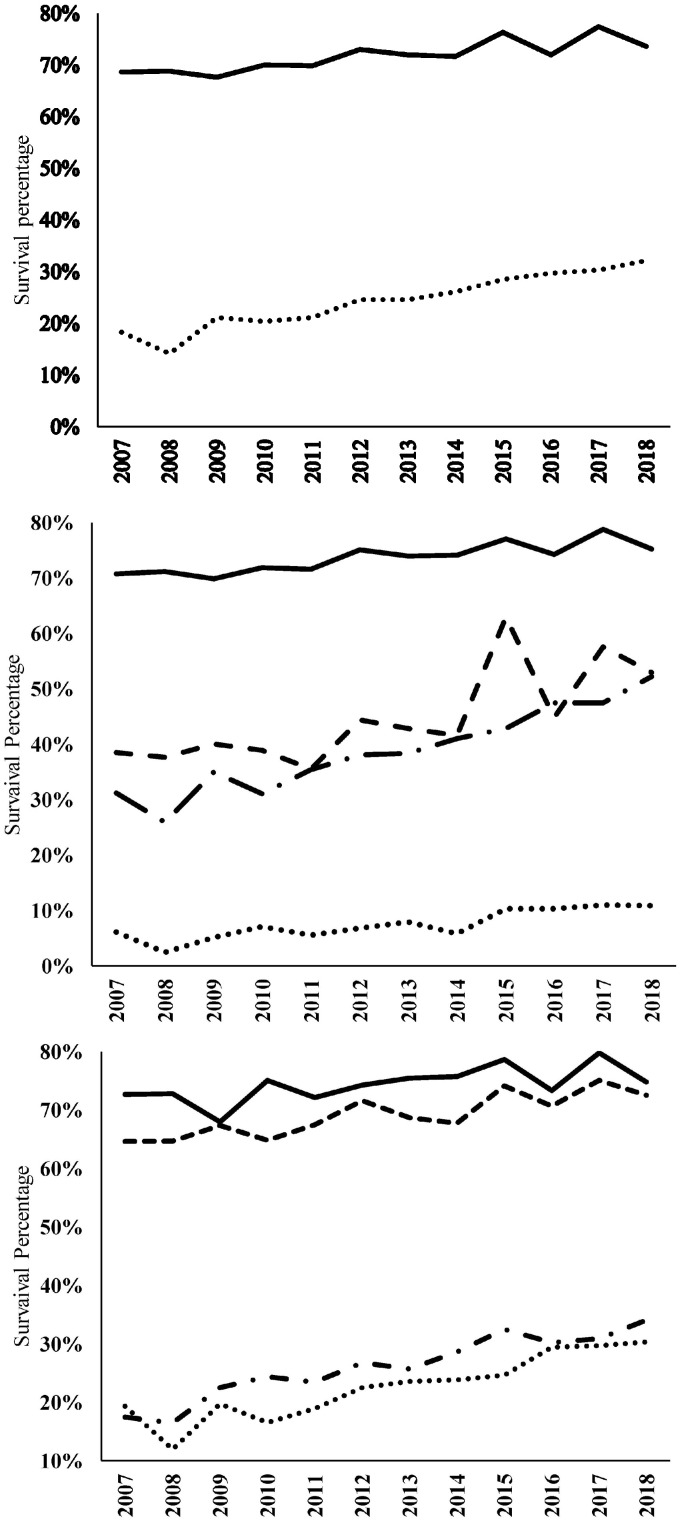
**Annual survival trends in periviable infants during the study period 2007–2018**. The upper panel represents the trend over time for survivability in preterm babies with completed 24 weeks (Solid line) and <24 weeks (Dotted s line). The middle panel represents the trends over time for survivability according to a combination of GA & BW: completed 24 weeks & >500 g (solid line), completed 24 weeks &<500 g (dashed line), <24 weeks & >500 g (dash-dot line), and <24 weeks &<500 g (dotted line). The lower panel represents the trend over time for survivability according to the combination of GA & sex: completed 24 weeks & female (solid line), completed 24 weeks & male (dashed line), <24 weeks & female (dash-dot line), and <24 weeks & male (dotted line).

Trend analyses were conducted for survival in periviable infants. In infants with GA <24 weeks, survival started at 18.4% in 2007 and was 31.9% by the year 2018 (Figure 1). The year-to-year change in survival rate was significant (*Z* = 9.438, *p*<0.0001). For infants born with completed 24 weeks, survival started at 68.4% in 2007 and was 73.3 % by the year 2018. The year-to-year change in survival was significant (*Z* = 3.3, *p* = 0.001).

Annual trends for survivability over time in the four subgroups of combined GA and BW are presented in Figure 1. Survival trends over time in the four groups were as follows: In infants < 24 weeks and ≤500 g, survival changed from 6.1% to 10.9% (*Z* = 2.26, *p*<0.02), in infants <24 weeks and >500 g, survival changed from 31.3 % to 52.2% (*Z* = 8.21, *p*<0.001), in infants with completed 24 weeks and <500 g, survival changed from 38.4% to 52.9 (*Z* = 5.81, *p*<0.001), and in infants with completed 24 weeks and >500 g, survival changed from 70.7% to 75.3% (*Z* = 3.68, *p*<0.008).

When combining GA with sex, there were 4 groups (<24 weeks/female, <24 weeks/male, completed 24 weeks/female, and completed 24 weeks/male). The trends for survival over time were statistically significant in each of these four GA/sex groups. Survival in the two female groups was higher than in the corresponding two male groups. (Figure 1).

**Figure 2:**
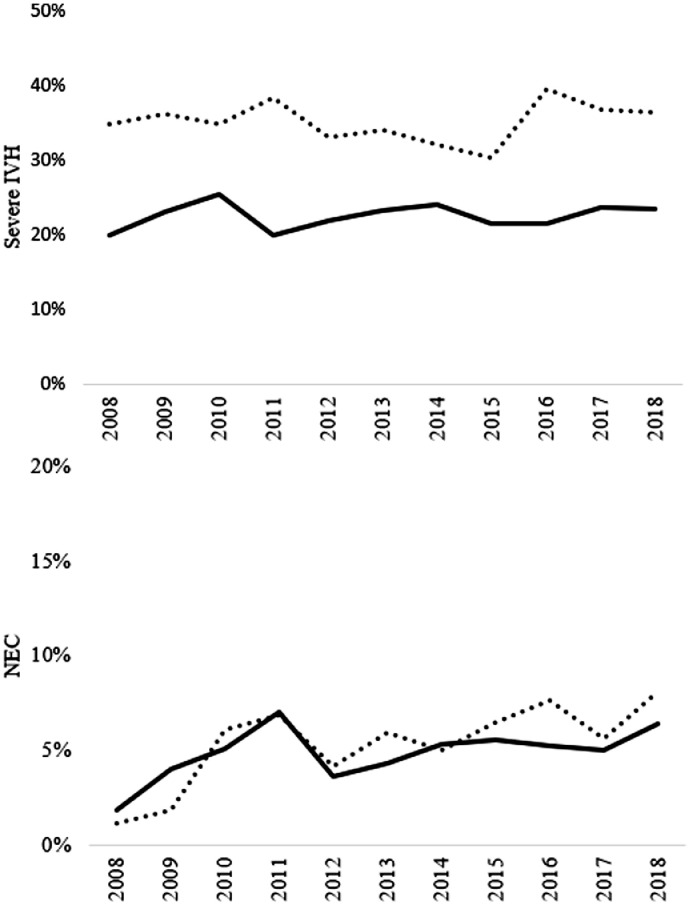
**Trends over time for severe intraventricular (IVH) hemorrhage and necrotizing enterocolitis (NEC) during the study period 2007–2018**. The upper panel represents the annual trend over time for severe IVH and the lower panel represents trend over time for NEC in infants with completed 24 weeks (solid line) and <24 weeks of gestation (dotted line).

Figure 2 represents annual trends for the prevalence of the two major morbidities in survived preterm infants, severe IVH and NEC. There was no evidence for a change in the prevalence of severe IVH over the years in infants with GA < 24 weeks (*Z* = 0.75, *p* 0.45) and in infants with completed 24 weeks (*Z* = 1.13, *p* = 0.25). Annual prevalence of NEC increased significantly over the years in infants with GA <24 weeks (*Z* = 5.77, *p* <0.001) and in infants with completed 24 weeks of gestation (*Z* = 2.54, *p*< 0.01).

Length of hospital stay and total charges for survived infants with GA < 24 weeks and completed 24 weeks of gestation are shown in Table 2. The Median (interquartile range) for the length of stay was 113 (55) and 114 (40) days, respectively. Median (interquartile range) charges for survived infants < 24 weeks and completed 24 weeks were $1,050,953 (786,989) and $ 1,016,751 (696,767), respectively.

**Table 2:** Length of stay and total charges for surviving infants with GA ≤ 24 weeks.

	Preterm infants < 24 weeks	Preterm infants 24 weeks	*P*-value
Length of stay (Days)	118 (99–141)	110 (95–130)	<0.001
Total charges ($)	926,105 (597,692–1,372,484)	895,137 (604,737–1,282,970)	<0.001

Data are expressed in median (interquartile range). Mann Whiney test was used.

## Discussion

The study presented the national survival of 24.1% and 71.6% for preterm infants with GA <24 weeks and completed 24 weeks, respectively. Female infants had higher survival rates than male infants. Infants of minority races (Black and Hispanic) had higher survival rates than White infants. Birth weight category was a significant factor associated with survival in periviable preterm infants. There was a trend for higher survival in infants with GA <24 weeks and in infants with completed 24 weeks. There was no evidence of long-term neurological changes. Prevalence of NEC has increased significantly in surviving periviable infants.

The majority (71.6%) of infants born with GA of 24 weeks survived, whereas only 24.1% of infants <24 weeks survived. The lower survival in infants < 24 weeks could be attributed to one of two factors. First, infants at this extreme degree of immaturity were biologically unable to adapt to the extrauterine environment while using current technologies. Second, caregivers, along with parents, might have agreed on providing comfort care only given the high mortality and risk of severe complications associated with resuscitating infants born at the limit of viability.^5^ In the US, there appears to be a significant discordance among providers regarding their preferred actions at the delivery of 23 and 22 weeks of gestation.^8^ The difference in providers' attitudes towards resuscitation interplays with the variation in outcome literature of these infants. Providing a national benchmark for the survival of periviable infants can be a helpful tool for parents and caregivers to exercise when making decisions.

Survival for infants born at 24 weeks changed over the study years from 68.4% to 73.3% and survival for infants born <24 weeks changed from 18.4% to 31.9%. These changes over time could be mostly attributed to recent practice changes in resuscitating infants at periviable gestational age. Changes in guidelines for management and resuscitation of infants at the edge of viability would also contribute to improved survival, as many centres have adopted new guidelines for rescuing infants with GA of 22 and 23 weeks.^8^ The decision to resuscitate a periviable infant is a shared resolution among caregivers and parents. Therefore, providing the findings of this study is an essential tool to formulate an informed team decision. Given the paucity of periviable infants, estimated at 0.28% of births, it is almost impossible for a centre to create its own outcome data for this population, and it becomes more meaningful to utilize a national benchmark for reference purposes.

Infants of Hispanic and Black ethnicity had higher survivals than White in the GA <24 weeks (aOR = 1.33, CI: 1.31–1.36, *p* < 0.001 and aOR = 1.29, CI: 1.27–1.32, *p* < 0.001, respectively) and in the GA of completed 24 weeks (aOR = 1.24, CI: 1.20–1.32, *p* < 0.001 and aOR = 1.27, CI: 1.22–1.30, *p* < 0.001, respectively). Although the data on ethnic differences in infants ≤24 weeks of gestation are scarce, previous studies on overall survival of any premature infant showed higher perinatal and neonatal survival among the White population.^9^^,^^10^ There is no clear explanation for this paradoxical effect of race on infants born at the limit of viability compared to premature infants of older GA. One of the explanations could be the ethnic differential in choosing delivery sites. Studies showed a tendency for the Hispanic and Black populations to deliver at high-risk centres while the White population is more inclined to deliver in community-based centres.^9^^,^^11^ As there is no current data on ethnic differences in early pregnancy miscarriages, in-utero selection could be another possibility.^12^

Birth weight is an essential factor for survivability when combined with GA. Several studies used BW as a surrogate for prematurity and provided outcomes for infants based on BW only.^13^ Other studies provided outcomes of preterm infants at different GA without considering BW.^3^ However, the current study provides the benchmark outcomes for periviable infants while combining GA and BW categories. Interestingly, infants <24 weeks with BW>500 g have a survival rate close to infants with completed 24 weeks and BW ≤500 g (38.9% and 45.1%, respectively). There is a more than four-fold increase in survival at the same GA when BW is >500 g; for example, infants <24 weeks had a survival rate that could be 38.9% or 7.4% depending on whether BW is >500 g or ≤500 g, respectively. Given the fact that intrauterine growth restriction is not uncommon in preterm infants,^14^ it is helpful for caregivers to inquire about the estimated fetal BW when approaching prenatal counselling to provide parents with accurate estimates for survival. Meanwhile, it should be acknowledged that the current study used BW measured postnatally which may not necessarily be identical to estimated fetal weight before birth.

The median charge for the care of an infant born at GA <24 weeks is close to $1M ($926,105), which translates to around $7B per year should all infants born at this age survive. These charges are limited to hospitalization at the birthing centre and do not include any long-term burden on the healthcare system. The current study does not intend to discuss healthcare policies, although it provides estimates that could be used by lawmakers and financial planners.

The prevalence of severe IVH in infants born at ≤ 24 weeks did not change over the study years 2007–2018 despite a significant trend for increased survival in this population. This finding should be cautiously interpreted as the study did not offer any data on long-term outcomes. The extent of vulnerability of the brain to the environment is unclear, and would not be reassured without assessing their long-term neurodevelopmental outcome. The trend for NEC showed a higher prevalence over time. This finding may imply the need to develop a feeding strategy and milk formula for periviable infants distinct from strategies and formulas currently used in more mature infants.

This study has the strength of being the largest reported in the literature with a sample that exceeds 44 million infants and represents the entire US, thereby accounting for all variations in practice and experiences that are observed in currently available studies. In addition, the study could provide the national trend over the years for outcomes. The study inherited some limitations; errors or omissions may occur when entering diagnostic codes (ICD-9 and ICD-10). However, the likelihood of these diagnostic errors is rare; especially, the NIS has developed validation methods to ensure the integrity of the data. Nonetheless, it is almost impossible to have errors in the dataset related to survival and mortality. The study utilized a national dataset to investigate the survival of infants concerning two system-based social factors: insurance type and household income; in addition to major infant demographics such as BW, sex, and ethnicity/race. Thus, it did not assess the association of survival with multiple clinical exposures related to maternal health such as maternal age, diabetes and anaemia; and the impact of these clinical factors is well-described in the literature.^15^, ^16^, ^17^ However, other measured or unmeasured confounding variables could exist and may change the reported associations. This dataset is limited to the inpatient setting; therefore, long-term follow-up after hospital discharge is unavailable.

## Conclusions

This study used the US National database and showed a national change in survival rates of infants born at periviable GA over time in the years 2007–2018. Female sex had higher survival than males, and ethnic minorities had higher survival than White. BW category is a fundamentally important factor associated with >4 folds higher survival in these infants. This study provides national benchmarks that can be used during prenatal counselling. Further studies directed towards describing long-term assessment of periviable infants are needed. Until then, the results of this study should be cautiously interpreted as long-term outcomes for this population are unknown.

## Contributors

IQ conceptualized and designed the study, conducted the analysis, drafted the initial manuscript, and reviewed and revised the manuscript. MAAF drafted the initial manuscript, reviewed and revised the manuscript. NA and MOK drafted the initial manuscript, reviewed and revised the manuscript. MAM critically revised the manuscript and revised statistical analyses and approved the final draft of the manuscript. HA conceptualized and designed the study, interpreted the analysis, drafted the initial manuscript, and reviewed and revised the manuscript. IQ and HA directly accessed and verified the underlying data reported in the manuscript. All authors approved the final manuscript as submitted and agree to be accountable for all aspects of the work.

## Data sharing statement

The study utilized HCUP data which is publically available upon request.

## Declaration of interests

We declare no competing interests.

## Appendix Supplementary materials

Image, application 1
