# Supplementary material for: Survival of infants born at periviable gestation: The US national database
Source: Lancet Reg Health Am. 2022 Jul 25;14:100330. doi: 10.1016/j.lana.2022.100330 (PMC9903864; doi:10.1016/j.lana.2022.100330)
Supplement: Supplementary file 1 [file mmc1.docx]

**Table S1·** ICD-9 and ICD-10 codes used for the study

| **ICD9** | **ICD10** |
| --- | --- |
| 765·01 weight less than 500 grams | [P07·01](https://www.icd10data.com/ICD10CM/Codes/P00-P96/P05-P08/P07-/P07.01) weight less than 500 grams |
| [765·22](http://www.icd9data.com/2015/Volume1/760-779/764-779/765/765.22.htm) and 765·21 used for 24 and less than 24 weeks of gestation, respectively· | [P07·22](https://www.icd10data.com/ICD10CM/Codes/P00-P96/P05-P08/P07-/P07.22), [P07·23](https://www.icd10data.com/ICD10CM/Codes/P00-P96/P05-P08/P07-/P07.23) and [P07·21](https://www.icd10data.com/ICD10CM/Codes/P00-P96/P05-P08/P07-/P07.21) used for gestational age 24, 23 completed weeks and < 23 weeks, respectively |
| 777·52 Stage 2 necrotizing enterocolitis in newborn | [P77·2](https://www.icd10data.com/ICD10CM/Codes/P00-P96/P76-P78/P77-/P77.2) Stage 2 necrotizing enterocolitis in newborn |
| [777·53](http://www.icd9data.com/2015/Volume1/760-779/764-779/777/777.53.htm) Stage 3 necrotizing enterocolitis in newborn | [P77·3](https://www.icd10data.com/ICD10CM/Codes/P00-P96/P76-P78/P77-/P77.3) Stage 3 necrotizing enterocolitis in newborn |
| intraventricular hemorrhage: Grade 3: 772·13· Grade 4: 772·14 | intraventricular hemorrhage: Grade 3: [P52·21](https://www.icd10data.com/ICD10CM/Codes/P00-P96/P50-P61/P52-/P52.21)· Grade 4: [P52·22](https://www.icd10data.com/ICD10CM/Codes/P00-P96/P50-P61/P52-/P52.22) |
